# Potential Therapeutic Implications of Caffeic Acid in Cancer Signaling: Past, Present, and Future

**DOI:** 10.3389/fphar.2022.845871

**Published:** 2022-03-09

**Authors:** Manzar Alam, Ghulam Md Ashraf, Kayenat Sheikh, Anish Khan, Sabeeha Ali, Md. Meraj Ansari, Mohd Adnan, Visweswara Rao Pasupuleti, Md. Imtaiyaz Hassan

**Affiliations:** ^1^ Centre for Interdisciplinary Research in Basic Sciences, Jamia Millia Islamia, New Delhi, India; ^2^ Pre-Clinical Research Unit, King Fahd Medical Research Center, King Abdulaziz University, Jeddah, Saudi Arabia; ^3^ Department of Medical Laboratory Sciences, Faculty of Applied Medical Sciences, King Abdulaziz University, Jeddah, Saudi Arabia; ^4^ Department of Computer Science, Jamia Millia Islamia, New Delhi, India; ^5^ Chemistry Department, Faculty of Science, King Abdulaziz University, Jeddah, Saudi Arabia; ^6^ Center of Excellence for Advanced Materials Research, King Abdulaziz University, Jeddah, Saudi Arabia; ^7^ Centre for Pharmaceutical Nanotechnology, Department of Pharmaceutics, National Institute of Pharmaceutical Education and Research, SAS Nagar Mohali, India; ^8^ Department of Biology, College of Science, University of Hail, Hail, Saudi Arabia; ^9^ Department of Biomedical Sciences and Therapeutics, Faculty of Medicine and Health Sciences, Universiti Malaysia Sabah, Kota Kinabalu, Malaysia; ^10^ Department of Biochemistry, Faculty of Medicine and Health Sciences, Abdurrab University, Pekanbaru, Indonesia; ^11^ Centre for International Collaboration and Research, Reva University, Bangalore, India

**Keywords:** caffeic acid, cancer, anti-cancer, anti-oxidant activity, bioavailability, clinical trials

## Abstract

Caffeic acid (CA) has been present in many herbs, vegetables, and fruits. CA is a bioactive compound and exhibits various health advantages that are linked with its anti-oxidant functions and implicated in the therapy and prevention of disease progression of inflammatory diseases and cancer. The anti-tumor action of CA is attributed to its pro-oxidant and anti-oxidant properties. CA’s mechanism of action involves preventing reactive oxygen species formation, diminishing the angiogenesis of cancer cells, enhancing the tumor cells’ DNA oxidation, and repressing MMP-2 and MMP-9. CA and its derivatives have been reported to exhibit anti-carcinogenic properties against many cancer types. CA has indicated low intestinal absorption, low oral bioavailability in rats, and pitiable permeability across Caco-2 cells. In the present review, we have illustrated CA’s therapeutic potential, pharmacokinetics, and characteristics. The pharmacological effects of CA, the emphasis on *in vitro and in vivo* studies, and the existing challenges and prospects of CA for cancer treatment and prevention are discussed in this review.

## Introduction

Caffeic acid (CA) is a phenolic derivative generally found in green tea, red wine, fruits, vegetables, and coffee (Chen et al., 2019; Meinhart et al., 2019; Zhang et al., 2019). CA (3,4-dihydroxycinnamic acid) exhibits anti-bacterial and anti-inflammatory effects and participates in significant functions of the human system (Kępa et al., 2018; Bounegru and Apetrei, 2020). It demonstrates anti-cancer, anti-oxidant, anti-proliferative, and anti-inflammatory properties. CA plays a pro-oxidant role in tumor cells and an anti-oxidant role in healthy cells. The oxidative DNA injury induced by the pro-oxidant property and its downstream pathway stimulate cell death by apoptosis (Kanimozhi and Prasad, 2015). CA has been frequently found as quinic acid ester called chlorogenic acid (Chen and Ho, 1997; Verma and Hansch, 2004; Silva et al., 2014).

CA plays in the protection machinery of plants against infections, predators, and pests, inhibiting the growth and survival of insects, fungi, and bacteria (Tošović, 2017). Polyphenols are organic compounds distinguished by huge manifolds of phenol structural parts that perform as the bases of exclusive chemical, biological, and physical functions to individual constituents of the class. This significant structural variety deeply influences their bioavailability (Birková et al., 2020). The defensive effect of CA on the human system is elucidated because of its anti-oxidant functions that are endorsed for its chemical structure. The chemical features of CA molecules permit the removal of free radicals and inhibit reactive oxygen species (ROS) formation; thus, it has useful impacts on human health (Birková et al., 2020).

CA and its derivatives have been identified with anti-oxidant, anti-viral, anti-inflammatory, and anti-cancer activities (Prasad et al., 2011; Yang W. S. et al., 2013).; Yang et al., 2013b). CA acts as an inhibitor of low-density lipoprotein oxidative alteration that is considered engaged in the pathogenesis of atherosclerosis (Wang and Yang, 2012). CA blocks STAT3 action, and this, in turn, down-triggers HIF-1α action. It is a promising inhibitor of STAT3 and represses cancer angiogenesis *via* blocking the action of STAT3 and the expression of VEGF and HIF-1α (Jung et al., 2007). Moreover, the mRNA levels of iNOS, COX-2, and TNF-α were less regulated *via* CA. CA strongly suppresses the nuclear translocation of AP-1 member proteins (Yang W. S. et al., 2013) and may simultaneously repress the activation of NF-κB, NFAT, and AP-1 (Feng et al., 2005).

Based on the broad effects of CA, here we discuss its therapeutic potential in cancer by emphasizing its function in cancer signaling. This review focuses on CA’s chemical and pharmacological effects by representing its mechanism of action, bioavailability, and pharmacokinetic characteristics for expressing the promising therapeutic function in tumors, with emphasis on *in vitro* and *in vivo* studies. To conclude, CA’s current challenges and prospects for cancer management are also discussed.

## Structural Features of Caffeic Acid

Phenolic compounds offer defense against diseases by regulating cellular mechanisms at different levels, such as enzyme inhibition, protein phosphorylation, and alteration of gene expression (Naz et al., 2017; Naz F. et al., 2018; Naz H. et al., 2018; Ishtikhar et al., 2018; Mohammad et al., 2019; Mohammad et al., 2020; Kasprzak-Drozd et al., 2021). An enhancement in phenolic compounds may change their health advantages (Saibabu et al., 2015; Anwar et al., 2016). However, more than 8,000 phenolic compounds might be categorized into two major groups, such as flavonoids and non-flavonoids (Kumar and Pandey, 2013).

Phenolic acids (PAs) are non-flavonoid phenolic compounds, including a single phenyl group alternated through a carboxylic and one or more OH groups (Leonard et al., 2021). PAs are again categorized by the extent of the chain, which encloses the carboxylic group, including hydroxycinnamic acids (HCs), hydroxyphenyl acids, and hydroxybenzoic acids (Has). HC has a C6–C3 fundamental skeleton. The existence of a CH_2_ = CH–COOH set in cinnamic acids guarantees a superior anti-oxidant capability than the COOH set in benzoic acid. Hence, one of the main HCs is CA (Göçer and Gülçin, 2011; Vinayagam et al., 2016; Filipe et al., 2018). Moreover, the potential therapeutic prospective of CA examinations has exhibited that the clean shape of CA has the accessibility to be absorbed in the intestines and consequently interfaces with the intention tissue (Sato et al., 2011).

## Bioavailability and Metabolism of Caffeic Acid

CA’s partition coefficient fluctuates between 1.0 and 1.3, and its molecular mass is 180.16 g/mol (Wu et al., 2007; Kudugunti et al., 2010). Monocarboxylic acid transporters are responsible for CA absorption in the gastro-intestinal tract (Konishi et al., 2004; Konishi and Kobayashi, 2004). CA metabolism is also linked to the gut microbiota. CA undergoes decarboxylation under an anaerobic condition which passes *via* bacteria with the production of an analog [3-(3-hydroxyphenyl)-propionic acid] and thus exhibits better anti-oxidant action as compared to CA (Shen et al., 2020). After absorption, CA undergoes widespread metabolic pathways in the liver and kidney (Ito et al., 2005; Lafay et al., 2006). CA has disclosed an excellent safety summary in phase 1 clinical trial.

Hydrolyzation is an incredibly chief step in the human metabolism of CA in the intestine because of esterases, enzymes that are able to hydrolyze chlorogenic acid to make CA. CA is found in its ester in extremely tough foods to get absorbed. The ingestion of CA starts in the stomach, where a little amount is absorbed. Following the action of microbial esterases in the colon, CA gets sliced in free appearance and 95% gets absorbed *via* the intestinal mucosa (Birková et al., 2020) by active transport induced through monocarboxylic acid transporters. Hence, the highest CA plasma concentration has been detected to reduce 1 h after food ingestion. The detoxification method instantly creates more hydrophilicity after absorption, decreasing its toxic effect and facilitating its removal. The small intestine is the probable location of feruoylquinic acid cleavage into CA and ferulic acid, CA metabolism into 3-O-sulfate and 4-O-sulfate, as well as CA methylation, resulting in the formation of isoferulic acid followed by 3-O-sulfation and glucuronidation. CA is emitted mainly by urine, with calculated urinary secretion being between 5.9 and 27% (Olthof et al., 2001; Manach et al., 2004; Espíndola et al., 2019).

## Role of Caffeic Acid in Human Physiology

CA is HA with a C6–C3 skeleton and with a transethylene wire connecting an aromatic ring with a carboxylic acid (Magnani et al., 2014; Silva et al., 2014). Plant CA biosynthesis occurs *via* a pathway which makes AAA from glucose (Silva et al., 2014; Rodrigues et al., 2015). Beginning with shikimic acid, it undergoes three enzymatic reactions. The first reaction is shikimate kinase-induced phosphorylation, followed by the conjugation of phosphoenolpyruvate induced by 5-EPSP synthase and lastly by chorismate synthetase, producing one of the vital conciliator metabolites of this signaling, chorismic acid (Silva et al., 2014; Ishtikhar et al., 2015; Rodrigues et al., 2015). Chorismic acid is converted by chorismate mutase into prephenic acid.

Furthermore, L-phenylalanine production is induced as a coenzyme by deamination through pyridoxal 5-phosphate and as an electron exchanger through nicotinamide adenine dinucleotide (Silva et al., 2014; Rodrigues et al., 2015). The deamination of L-phenylalanine through phenylalanine ammonia lyase forms cinnamic acid, which is transformed into p-coumaric acid through C4H and CA through the enzyme C3H (Rodrigues et al., 2015). CA is attained from plants by solvent extraction at the highest temperature, though its yield is extremely low, involving huge amounts of botanical substance to obtain a considerable yield (Lin and Yan, 2012; Rodrigues et al., 2015). This compound can be achieved in huge quantities through organic synthesis (Touaibia and Guay, 2011). Genetics alter microorganisms—for example, *Escherichia coli* strains (Kawaguchi et al., 2017; Hernández-Chávez et al., 2019).

There are several beneficial effects of CA and its derivatives, including anti-bacterial (Matejczyk et al., 2018; Mitani et al., 2018), anti-viral (Langland et al., 2018; Shen et al., 2018), anti-oxidant (Shiozawa et al., 2018; Kfoury et al., 2019), anti-inflammatory (Zaitone et al., 2019; Lima et al., 2020), immune-stimulatory (Krifa et al., 2013; Coleman et al., 2016), antidiabetic (Chiou et al., 2017; Bounegru and Apetrei, 2020), cardioprotective (Agunloye et al., 2019; Salau et al., 2021), anti-proliferative (Pelinson et al., 2019), hepatoprotective (Abdelhafez et al., 2018; Saleem et al., 2019), anti-cancer (Zeng et al., 2018; Martini et al., 2019; Pelinson et al., 2019), and so on. CA plays a central function in the human system because of its many beneficial effects. It can be found in pharmaceuticals (Katsarova et al., 2017; Li et al., 2017).

Several studies showed that high doses of CA might cause considerable side effects, which inhibit the implantation of embryos (Liu et al., 2019) or cancerous effects (Hagiwara et al., 1991). The information is verified; the small intestine absorbs a significant amount of CA, which goes into the bloodstream in a large proportion (Wang et al., 2017). CA can support the progression of squamous cell carcinomas in the kidneys and stomach of mice and rats (Hagiwara et al., 1991). A toxicity study of CA has been performed to understand the reproductive role and the progression of offspring in female mice. However, female mice have incessantly been exposed to different dosages *via* gavage in the 3-segment analysis. Two CA doses (5 and 150 mg/kg/day) were reported to affect embryo implantation when administered before the sixth day of gestation. Additionally, the CA dose of 150 mg/kg/day influenced the fetal weight to be achieved (Liu et al., 2019).

## Pharmacological Effects of Caffeic Acid

### Anti-cancer Properties

CA and its derivatives have been recognized for their anti-inflammatory, anti-bacterial, and anti-carcinogenic functions that could be associated with its anti-oxidant action (Genaro-Mattos et al., 2015). CA treatment has increased the ROS levels and changed matrix metalloproteinases (MMP) in ME-180 and HeLa tumor cells. Enhanced apoptotic morphological alterations have been observed in CA-treated cells in ME-180 and HeLa cells (Kanimozhi and Prasad, 2015). Hence, a pro-survival result of CA mediated by the NF-κB pathway has been expressed in lung tumor cells treated with paclitaxel (Lin et al., 2012). The accurate function of ROS in intracellular activity remains less understood and probably depends on particular situations.

New studies show that CA exerts anti-tumor properties by AMPK activation, and a mechanism has been identified in colon tumor cells *in vitro* (Murad et al., 2015). CA demonstrates a potent anti-tumor effect in the HT-1080 cell line, which might be utilized as an anti-cancer drug (Prasad et al., 2011; Alam et al., 2022). It prevented breast tumor cell proliferation, influencing cell cycle development and downstream effectors. The maximum impact of CA has been observed in MCF-7 cells, where it suppressed the proliferation and survival of breast tumor cells (Rosendahl et al., 2015).

The role of CA-targeting gene amplified in squamous cell carcinoma 1 (GASC1) has been established recently. GASC1 is a recently reported oncogene in various cancer types, including esophageal cancer (Sun et al., 2013; Jia et al., 2020). The anti-cancer effect of CA has been studied in advanced esophageal squamous cell cancer (ESCC), and clinical trials are being conducted (ClinicalTrials.gov identifier: NCT04648917). CA was used as a drug for thrombocytopenia when the patient received chemotherapy. A recent clinical trial has been done to see the effectiveness of oral CA tablets in managing primary immune thrombocytopenia. Scientists have observed a very effective role of CA in immune thrombocytopenia patients, with few and mild side effects, thus suggesting a potential therapeutic role of CA (Qin et al., 2015).

The anti-carcinogenic functions of CA have magnetized the consideration of the scientific society (Da Cunha et al., 2004; Damasceno et al., 2017; Sidoryk et al., 2018). Reports have revealed that the utilization of foods rich in CA causes a defensive action in carcinogenesis by inhibiting the creation of nitro compounds, the pathology’s chief inducers (Touaibia et al., 2011; Damasceno et al., 2017). However, these effects of CA are generally linked with its anti-oxidant (Stagos et al., 2012; Magnani et al., 2014) and pro-oxidant abilities (Li et al., 2000; Zhang et al., 2008), which are attributed to their chemical structure. Initially, the existence of free phenolic hydroxyls is probably to reduce the enthalpy of OH-bond dissociation that enhances the transfer speed of H atoms to peroxyl radicals and the number and location on the phenyl ring. Hence, the existence of a double bond in the carbon chain enhances the constancy of the phenolic radical (Son and Lewis, 2002; Touaibia et al., 2011; Magnani et al., 2014). Thus, chemical features connected with CA molecules permit the removal of free radicals, inhibiting the creation of ROS and the initiation of DNA oxidation of tumor cells (Silva et al., 2014; Sidoryk et al., 2018).

### Anti-oxidant Activity

CA is an anti-oxidant which may decrease the oxidative stress present in the body because of free radicals. Hence, oxidative stress is described as an inequity between the making of ROS and anti-oxidant protection (Birková et al., 2020). Consequently to this inequity, oxidative stress frequently results in the progression of many diseases in humans, including cancer (Ahmed et al., 2015; Bjørklund and Chirumbolo, 2017). Anti-oxidants inhibit the effects regulated *via* free radicals and oxidizing compounds (Soares et al., 2005). The grouping of CA with other products, including chlorogenic and caffeic acids, is explained by the high potent anti-oxidant action in different systems (Meyer et al., 1998; Fukumoto and Mazza, 2000). CA is considered as a potential photo-protective agent present in skincare products because of its anti-oxidant action (Yamada et al., 2006). Depending on the exposure time, wavelength, exposed area, and dose, UV radiation may cause premature skin aging, skin burns, skin cell DNA injury, and skin tumor (Scharffetter-Kochanek et al., 1997; Matsumura and Ananthaswamy, 2002).

Based on the mechanisms of CA, it acts in cancer *via* its promising anti-oxidant ability that inhibits the creation of ROS, thus decreasing oxidative stress, which is incredibly general in disease (Silva et al., 2014; Sidoryk et al., 2018). CA performs as a primary and pro-oxidant (secondary). A primary anti-oxidant performs *via* disrupting the creation of free radicals *via* preventing the chain reactions with a different molecule (Angelo and Jorge, 2007; Damasceno et al., 2017). This procedure happens when CA donates hydrogen/electrons for free radicals, changing them into thermodynamically constant products. Hence, these products present better constancy because of the electron delocalization in the aromatic ring of CA (Damasceno et al., 2017). A secondary anti-oxidant performs as a chelating agent and makes complexes with metals (copper and iron), preventing the decomposition of peroxides, decreasing the creation of free radicals and their assault on amino acids, lipids, and bases of DNA, and consequently evading the making of lesions and failure of cellular integrity (Angelo and Jorge, 2007; Damasceno et al., 2017). However, CA has a huge potential effect for decreasing metals because of its structural chemical features; the compound is vulnerable to auto-oxidation and oxidation caused *via* other biological agents (Medina et al., 2012; Damasceno et al., 2017).

### Pro-oxidant Activity

The utilization of foods rich in CA has been revealed to protect against carcinogenesis due to its anti-oxidant and pro-oxidant functions. CA displays pro-oxidative roles in cancer cells, which are correlated with oxidative DNA injury and, pursued by its consequent pathway, the induction of cell death in tumor cells (Birková et al., 2020). This anti-tumor effect of CA by pro-oxidative functions has been first identified by scientists in 2015. They detected enhanced apoptotic morphological alterations in tumor cells treated with CA, where CA enhanced the lipid peroxidation (LPO) markers in ME-180 and HeLa cells. Hence, they detected elevated levels of ROS and modified MMP (Kanimozhi and Prasad, 2015). CA may turn into a pro-oxidant by its capability to chelate metals like copper and stimulate LPO, causing injury on the DNA of tumor cells through oxidation or creation of covalent adducts with DNA (Zheng et al., 2008; Damasceno et al., 2017). CA holds the capability to cap the endogenous Cu ions of human lymphocytes to form CA-Cu (II) (Zheng et al., 2008). CA undertakes deprotonation relative to Cu, producing an oxygen center by high electronic density (Zheng et al., 2008; Damasceno et al., 2017). However, this complex ensures oxygen to make the semiquinone radical anion with Cu (I) (Zheng et al., 2008; Damasceno et al., 2017). CA deprotonation takes place to form a phenoxide wherever the Cu (I) ion should be bound as a bidentate linker (Zheng et al., 2008; Damasceno et al., 2017). Pro-oxidant acts play an anti-tumor effect due to the induction of cell death in the cancer cells (Yang et al., 2012).

### Anti-inflammatory Properties

CA exhibits a cardio-protective effect against hypercoagulability, dyslipidemia, inflammation, and oxidative stress in diabetic mice. The dietary supplementation of CA and ellagic acid increased the levels of lipid metabolism and glycemic control in diabetic mice (Birková et al., 2020; Sharifi-Rad et al., 2020). Eventually, both compounds explained the anti-inflammatory, anti-oxidative, and anti-coagulatory defense for the heart of diabetic mice (Chao et al., 2009). CA might defend the cardiac tissue against diabetes-linked hypercoagulability, dyslipidemia, inflammation, and oxidative stress. The inflammatory reaction in the brain is a coordinated regulatory machinery of specialized cells in the CNS known as microglial cells. However, the activation of cells under pathological conditions was revealed to contribute to the progression of numerous neurodegenerative disorders. The discharge of pro-inflammatory mediators in the brain stimulated through various stimulants, including Aβ, was exhibited to account for the inflammatory constituent of neuronal loss in Alzheimer's disease (AD) (Ito et al., 1998; Verri et al., 2012; Prokop et al., 2013).

CA and its derivatives possess anti-inflammatory effects and thus are implicated in AD through these agent candidates. In addition, the activation of Nrf2 has been explained for inhibiting inflammatory gene expression (Chen et al., 2006; Alam et al., 2021a) by signaling the crosstalk linking the HO-1 (Kapturczak et al., 2004). Kim *et al*. (Kim and Jang, 2014) have illustrated that the Nrf2-induced HO-1 introduction of CAPE is correlated with its anti-inflammatory and anti-oxidant mechanisms. The anti-inflammatory result of other CA esters in microglial cells was connected to the induction of HO-1 (Lu et al., 2013; Alam et al., 2021c). Hence, their multifunctional results indicate the relationship between the CA derivatives’ anti-oxidant and anti-inflammatory effect and their therapeutic potential for AD. Liang *et al*. (2015) have confirmed that the enhanced NF-κB, p65, and 5-LOX expression correlated with the global cerebral ischemia–reperfusion neuronal injury and memory loss in rats is inverted through CA (10–50 mg/kg) treatment. CA (50 mg/kg) also improved neuronal loss and infarct volume 24 h after ischemia (Zhou et al., 2006). Together with the common anti-inflammatory effect of CA derivatives, these effects are all pertinent mechanisms that might feature anti-AD potential.

### Induction of Apoptosis

CA induces apoptosis through inhibiting Bcl-2 action, resulting in the liberation of cyt-*c* and the consequent activation of caspase-3, representing the fact that CA stimulates apoptosis by the intrinsic apoptotic pathway (Chang et al., 2010; Alam et al., 2019; Alam et al., 2021b). Their anti-oxidant results are arbitrated through modulating pathways, including MAPK, NF-κB, and Akt. Furthermore, they stimulate cell cycle arrest and increase cell death in tongue, neck, and mouth cancer (Srinivasulu et al., 2018). In Ht-29 cells, 5-caffeoylquinic acid and CA decreased cell viability by endorsing specific cell cycle modifications and stimulating cell death in a time- and dose-dependent way (Murad et al., 2015; Anantharaju et al., 2016). CA demonstrated a vital function in inhibiting cancer progression by reducing cell viability and cell death induction. The treatment with CA caused the modulation of the cell cycle, prevention of colony formation, and alteration in caspase expression (Pelinson et al., 2019). CA attenuated cancer stem cell-like functions by the prevention of TGFβ-SMAD2 signaling induced *via* microRNA-148a *in vivo* as well as *in vitro* (Li et al., 2015). The contrast in the anti-tumor results of CA and CAPE showed the exposure time and dose-dependent capability of CAPE to be highly promising in the treatment of tumor cells with its action induced through stimulating cell death and cell cycle arrest in MDA-MB-231 cells (Kabała-Dzik et al., 2017) and decreasing the migration of MCF-7 (Kleczka et al., 2020).

### Inhibition of Vascularization and Invasiveness

**FIGURE 1:**
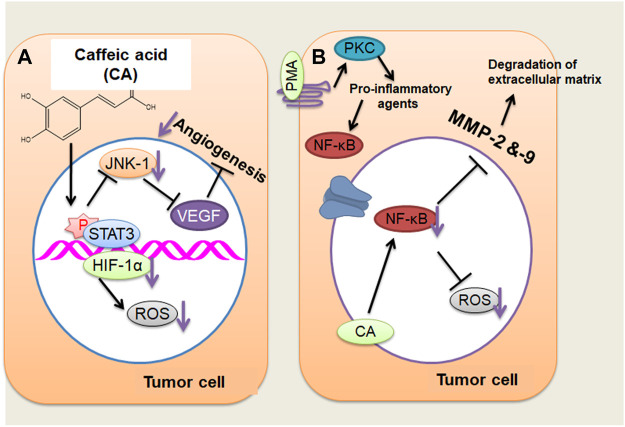
**(A)** Caffeic acid (CA) actions on the angiogenesis of cancer cells through decreasing the JNK-1 phosphorylation and reducing the HIF-1α activation that cause the decline of vascularization mediated by vascular endothelial growth factor. **(B)** CA may act on tumor cells by repressing MMP-2 and MMP-9 expressions, which, in turn, inhibits the activation of NF-κB stimulated through PMA (activating protein 1) in tumor cells, thus reducing cancer invasiveness and growth. ↓, decrease. (Adapted from Espíndola et al., 2019)

CA efficiently blocked the VEGF-mediated proliferation and survival of retinal endothelial cells in a concentration-dependent way (Figure 1A). Additionally, the tube formation of cells and VEGF-mediated migration have been presented (Kim et al., 2009; Alam and Mishra, 2020). CA might also perform hepatocellular carcinoma (HCC) cells’ angiogenesis by decreasing the JNK-1 phosphorylation through the reduction of HIF-1α activation. This causes the decline of vascularization mediated through VEGF and represses cancer growth (Gu et al., 2016). HCC is an extremely vascularized tumor whose main distinctive characteristic is angiogenesis, and its major resource of blood supply is the hepatic artery (Dhanasekaran et al., 2016; Gu et al., 2016; Klungboonkrong et al., 2017). This cancer is rich in vascularization; hypoxia is very frequent because of the quick proliferation of cancer cells and, accordingly, the making of huge solid tumor masses, hindering and squeezing the blood vessels about it (Dhanasekaran et al., 2016; Gu et al., 2016; Klungboonkrong et al., 2017). Cancer cells seek to adapt to hypoxia through activating HIF-1 by the JNK-1 pathway, which stimulates numerous proangiogenic factors, including VEGF. When better expressed, VEGF causes extravasation of blood from the cancer blood vessels, leading to hepatic bleeding (Zhu et al., 2011; Gu et al., 2016), a significant factor for cancer survival (Zhu et al., 2011; Dhanasekaran et al., 2016; Gu et al., 2016; Klungboonkrong et al., 2017). CA drastically repressed the retinal neovascularization in oxygen-mediated retinopathy like the animal model of ROP with no retinal cytotoxicity (Kim et al., 2009).

An additional mechanism of action suggested that CA represses MMP-2 and MMP-9 expression in HCC. MMP-2 and MMP-9 are expressed in cancer cells that degrade the extracellular matrix (ECM) type IV collagen during tumor metastasis and invasion (Chung et al., 2004; Lee et al., 2008; Yeh et al., 2012; Pramanik et al., 2016). Phorbol 12-myristate 13-acetate (PMA) can stimulate PKC; once activated, it endorses the induction of pro-inflammatory cytokines, including IL-6 and TNF-a (Jiang and Fleet, 2012). These pro-inflammatory mediators stimulate the activation of NF-κB by c-Src/ERK/NIK/IKK (Touaibia et al., 2011; Kim et al., 2014; Alam and Mishra, 2021). NF-κB produces enhanced MMP-2 and MMP-9 expression that leads to the metastasis and invasion of hepatic cells through the degradation of ECM (Chung et al., 2004; Lee et al., 2008; Yeh et al., 2012; Pramanik et al., 2018). The repressive result of CA on MMP-2 and MMP-9 is connected with the obstruction of NF-κB activation, as identified in liver tumor cells stimulated *via* PMA, leading to a reduction in cancer invasiveness and growth (Chung et al., 2004; Lee et al., 2008; Alam et al., 2017) (Figure 1B). CA has been noted of its anti-oxidant results *via* repressing the making of ROS and superoxide dismutase and inhibiting tumor development and migration by reducing cell adhesion *via* a decreased connection to the ECM in A549 and HT29-D4 cells (Anantharaju et al., 2016; Zhu, 2016).

### Synergistic Effect of Caffeic Acid With Anti-cancer Agents

Combination therapy is the treatment and management move toward two or more agents/drugs with the target of achieving equivalent efficiency levels with minor toxicities and at doses lesser than normal and having superior influence with synergistic/additive effects (Chen et al., 2016; Bayat Mokhtari et al., 2017). A combined treatment approach with natural products might inhibit the source of acquired drug resistance, including chemotherapy. However, successful combinations of potent therapeutic drugs/agents with products may attain the desired conclusion but with lesser toxicity (Bukowska et al., 2015; Das et al., 2018). CA and Metformin (Met) was identified to have additive/synergistic effects while combined with anti-tumor therapies, mainly for HTB-34 cells (Tyszka-Czochara et al., 2017b). CA induces cytotoxicity by necrosis for SiHa cancer cells but, combined with Met and its cytotoxicity machinery, moved toward cell death without disturbing healthy human fibroblasts. However, the combination for controlling mitochondrial metabolism that stimulated ROS making in metastatic cancer cells has been found. Hence, incubating tumor cells with CA and Met caused a remarkable move to the G0 from the G1 stage. Studies continue to explore the mechanism of anti-cancer function of the combination of CA and Met (Tyszka-Czochara et al., 2017a).

**FIGURE 2:**
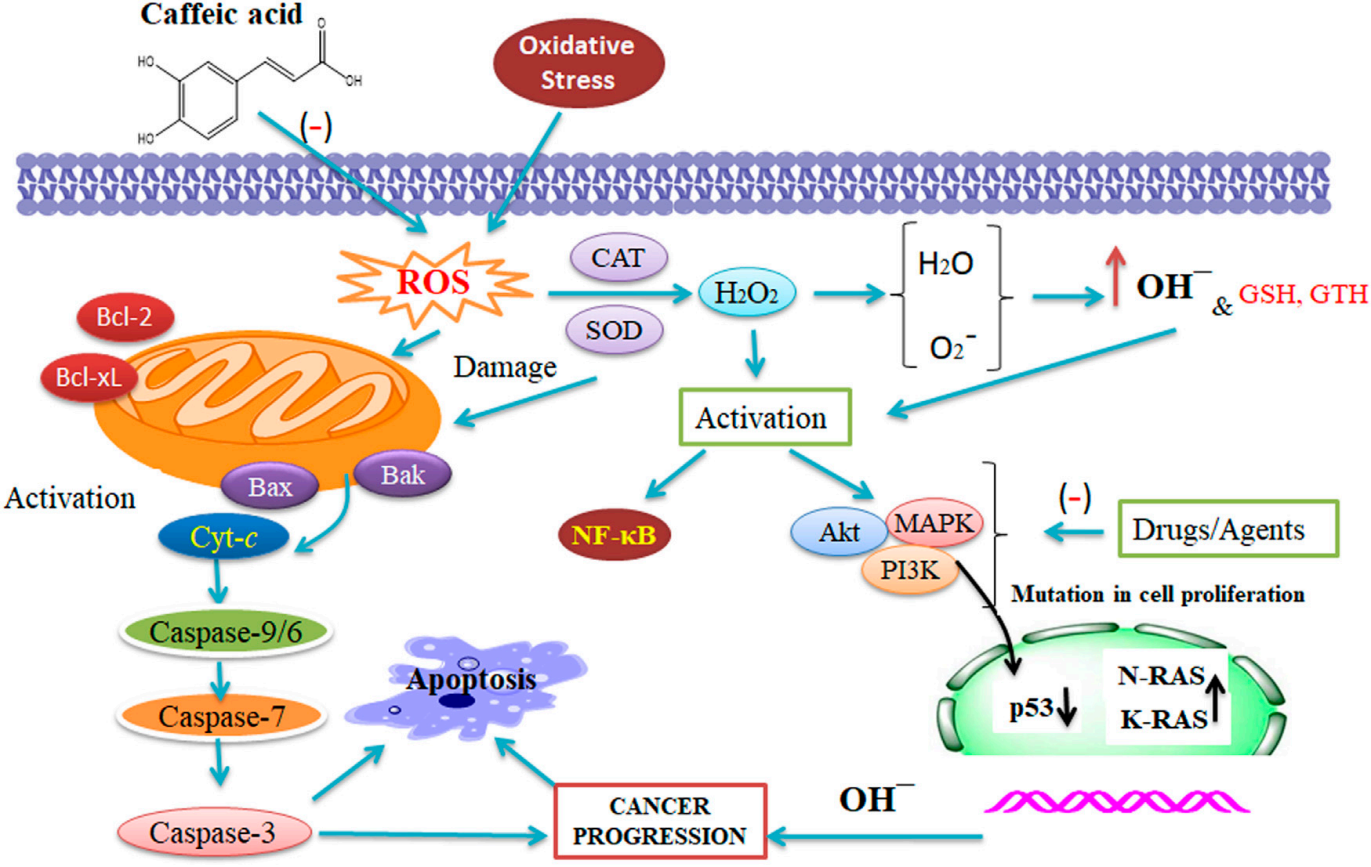
Synergistic action of caffeic acid (CA) with anti-tumor therapy that affects the reactive oxygen species, catalase, and superoxide dismutase. The combined result of CA and CAF reveals their potential against tumors and evaluates the molecular mechanisms of the combination with a several-target approach (Maity et al., 2021 and Alam et al., 2022). This figure was drown by ChemBioDraw.

CA and cisplatin illustrated a potent anti-tumor action in cancer. Furthermore, cisplatin-sensitive cells, while exposed to a combination therapy of 50 μM CA and 5 μM cisplatin, quickly enhanced the action of apoptotic cascade through enhanced caspase action (1.7 folds) in contrast to the administration of 5 μM cisplatin only. A study on A2780cisR cells confirmed that combining 5:50 μM (cisplatin/CA) increases the caspase action by 4:3 folds through 60% cell viability (Sirota et al., 2017). An analysis has been performed wherein the combination of CA with cisplatin has been checked to stop the resistance progression in tumor treatment. CA is an inhibitor of glutathione S-transferase and glutathione reductase that are the catalytic enzymes of GTH (Figure 2) (Siddik, 2003; Islam et al., 2017). Hence, there is a scope of testing the combined result of CA and CAF to reveal their potential therapeutic effect against tumor to evaluate the molecular mechanisms of the combination with a multi-target (Figure 2). However, CA combined with cisplatin enhanced its therapeutic effect, leading to the prevention of cell growth and survival of CaSki and HeLa cells that might be elucidated *via* a synergistic effect. Hence, this combination has been correlated with enhancing the expression of caspase-3, caspase-7, and caspase-9 (Koraneekit et al., 2018).

### Clinical Significance of Caffeic Acid

A study has explained that CAPE (50 μM) considerably enhanced the apoptosis induced *via* tumor necrosis factor-related apoptosis-inducing ligand (TRAIL) through the positive regulation of DR5 induced *via* CHOP in Hep3B HCC cells. However, TRAIL is a ligand with anti-tumor functions able to stimulate cell death in tumor cells (Mongkolsapaya et al., 1998; Dilshara et al., 2016). This act happens *via* its binding with DR5, which interrelates with Fas through recruiting caspase-8 and caspase-3 and stimulating cell death (Siegmund et al., 2001; Dilshara et al., 2016). CAPE found in the bee propolis extract potentiated TRAIL-induced apoptosis, motivating the CHOP protein expression being dependable for DR5 regulation (Dilshara et al., 2016). One experiment identified CAPE (30 μg/ml) to potentiate TRAIL-mediated cell death (30 ng/ml) by DR5 regulation through p38 and repression of JNK in SK-Hep1 cells (Yang SY. et al., 2013; Kim et al., 2014). However, the combination of TRAIL and CAPE has generated apoptosis by the intrinsic pathway and through the extrinsic pathway (Yang SY. et al., 2013). In intrinsic CAPE and TRAIL signaling, mitochondrial membrane depolarization stimuli have been enhanced, consequential in the liberation of cyt-*c* and the making of the apoptosome and resulting in the activation of apoptosis-stimulating caspase 9 (Nicholson, 1999; Yang SY. et al., 2013). Alternatively, CAPE and TRAIL endorsed p38 activation *via* the extrinsic signaling pathway by enhancing the expression of apoptosis, stimulating DR5, and blocking the JNK phosphorylation, which contributes to TRAIL resistance and, accordingly, reduced the DR5 expression (Lu et al., 2011; Yang SY. et al., 2013).

CA (1 mM) inhibited cell proliferation and survival in HCC cells extracted from marmots (Wilkins et al., 2017). Hence, the compound’s activity is connected with its participation in the failure of mitochondrial integrity, resulting in cyt-*c* liberation, apoptosome making, and caspase-9 activation, thus inducing apoptosis. CAPE (12.5 μM) blocked the invasion and MMP-2 and MMP-9 expression in SK-Hep1 cells that obstruct NF-κB (Lee et al., 2008). CA (200 μg/ml) blocked cancer regression and invasion in HepG2 and Huh7 cells *via* reducing pro-inflammatory cytokines, including TNF-a, IL-1b, and IL-8, and anti-inflammatory cytokines, including IL-10 (Guerriero et al., 2011). A neurotoxicity examination using PC12 cells treated with 10 μM Aβ (Sul et al., 2009; Lee et al., 2011; Kim J. H. et al., 2015; Yang et al., 2015) for 24 h confirmed that CA holds a cytoprotective effect in a dose-dependent way (10 and 20 μg/ml) when added 1 h earlier to Aβ (Sul et al., 2009). The pre-treatment of PC12 cell lines with chlorogenic acid (CGA) was proven to protect them from Aβ-mediated cell death together with attenuation of calcium levels and a decrease in the level of cell death-associated proteins such as caspase-3, Bax, and Bcl-2 (Lee et al., 2011). The defensive action of CA from Aβ (Sul et al., 2009; Lee et al., 2011; Kim J. H. et al., 2015; Yang et al., 2015) and LPS-mediated neuronal cell injury and neuronal inflammation has also been evaluated in C6 glial cells (Kim J. H. et al., 2015). However, the result exhibited a positive conclusion, but considering the greater dose (5 mM CA) utilized in the study, the result is not believed to be of therapeutic application until an action at a lesser dose is exhibited (Kim J. H. et al., 2015). In Aβ-mediated axonal atrophy in cultured cortical neurons of mice (Sul et al., 2009; Lee et al., 2011; Kim J. H. et al., 2015; Yang et al., 2015), CA 4-O-glucoside was shown to induce considerable axonal elongation results on Aβ-mediated atrophy (Sul et al., 2009; Lee et al., 2011; Kim J. H. et al., 2015; Yang et al., 2015).

The activity of CA was determined (100 mg/kg) on the structural alterations caused by HCC in the rat microbiota, showing that the compound decreases and alters the markers of liver damage during exposure to HCC, including transaminase, aspartate, alanine, aminotransferase, phosphatase, and total cholesterol. Hence, a possible machinery by CA actions is associated in the prevention of survival of malefic bacteria (Del Chierico et al., 2017; Zhang et al., 2017; Ali et al., 2021) and the introduction of the growth and survival of microbiota-beneficial bacteria during HCC progression (Li et al., 2016; Zhang et al., 2017). CA possesses anti-oxidant functions, making it capable of eliminating oxygen radicals and which facilitates the growth of beneficial bacteria, which are anaerobic and grow extremely without oxygen (Zhao et al., 2014; Zhang et al., 2017). Compounds possess an anti-microbial action in removing malefic bacteria of the microbiota, supporting the control of markers of liver injury (Pinho et al., 2015; Zhang et al., 2017).

CA (1 mM) improved the efficiency of Tris-acetate-EDTA (TAE) in rats with tumors. Hence, TAE is a therapeutic process utilized in patients with HCC to promote ischemia because of occlusion of the arterial blood supply, resulting in the obstruction of nutrients and oxygen for cancer (Johnson et al., 2016; Wilkins et al., 2017). The chief nutrient for HCC is lactate, which is created by glycolytic metabolism that is dependable for enhancing vascular growth factor expression in vasculogenesis (Mathupala et al., 2007; Wilkins et al., 2017). This effect is probably because of the anti-cancer, anti-inflammatory, and anti-oxidant properties that produce ROS and fragment DNA, which causes apoptosis in tumor cells (Wilkins et al., 2017). CA can stimulate the intrinsic pathway of cell death by changing the mitochondria’s membrane potential (Prasad et al., 2011; Wilkins et al., 2017).

CA is an extremely flexible compound with various biological actions impacting the human system, including anti-tumor, anti-oxidant, anti-microbial, anti-inflammatory (Verma and Hansch, 2004; Yang SY. et al., 2013; Genaro-Mattos et al., 2015). This information seems to favor its activity in the HCC, as *in vitro* and *in vivo* investigations already exhibited its act by numerous mechanisms of action in the battle against diseases, including ROS prevention (Silva et al., 2014; Sidoryk et al., 2018), angiogenesis (Gu et al., 2016), and repression of MMP-2 and MMP-9 (Chung et al., 2004; Gu et al., 2016), thus explaining the diversities in the effects found. Kim *et al*. (2015) examined the defensive capabilities of CA in an Aβ-injected (Sul et al., 2009; Lee et al., 2011; Kim J. H. et al., 2015; Yang et al., 2015) AD mouse model through the administration of 10–50 mg/kg/day for 2 weeks. CA, in a dose-dependent way, blocked LPO and nitric oxide creation in the kidney, liver, and brain, while this was contrasted with the Aβ-injected (Sul et al., 2009; Lee et al., 2011; Kim J. H. et al., 2015; Yang et al., 2015) healthy group. In kainic acid-mediated cognitive dysfunction in rats, CA demonstrated a considerable enhancement in memory presentation, oxidative stress parameters, and a mitochondrial role compared to the control group (Kumar et al., 2012). By utilizing the global cerebral ischemia–reperfusion damage analysis model in rats, a study (Liang et al., 2015) has examined the effect of CA on memory wherever the bilateral carotid artery has been occluded for 20 min as pursued *via* reperfusion. The analysis discovered that CA (10–50 mg/kg) noticeably decreased the escape latency, reassured hippocampal neuron injury, and enhanced the neuronal count compared to that in untreated rats.

### Pharmacokinetics of Caffeic Acid

CA occurs in esterified and free forms, indicating around 75–100% of the entire content of hydroxycinnamic acid in fruits (Lee et al., 1995). CA is tricky to be absorbed through the body (Lee et al., 1995; Scalbert and Williamson, 2000; Kolodziejczyk-Czepas et al., 2015). To be absorbed, this compound requires to be hydrolyzed *via* the colonic microflora in the intestine since human tissues and biological fluids do not have enzymes, known as esterases, able to hydrolyze the CGA to liberate CA (Lee et al., 1995; Scalbert and Williamson, 2000; Kolodziejczyk-Czepas et al., 2015). Accordingly, the pharmacokinetic procedure starts with the ingestion of CA inward in the stomach, after which a little part is absorbed. Hence, in the colon, the microbial esterases slice the ester piece of CA. It is absorbed through the intestinal mucosa (Oliveira and Bastos, 2011). The transmembrane run of CA into the intestinal cells is *via* active transport mediated through MCT (Oliveira and Bastos, 2011). The highest plasma concentration of the compound has been detected merely 1 h after ingestion of foods, including coffee, and subsequently, the plasma concentration quickly reduced, requiring reiterated doses every 2 h to sustain the high concentrations (Lee et al., 1995; Scalbert and Williamson, 2000; Kolodziejczyk-Czepas et al., 2015). Instantly after absorption, CA is subjected to three major procedures of enzymatic conjugation—methylation, sulphation, and glucuronidation—by the action of UDP-glucosyltransferases and catechol-o-methyltransferases. Hence, this creates a high level of hydrophilicity, decreasing its toxicity and assisting its exclusion (Oliveira and Bastos, 2011). The emission of CA (5.9–27%) takes place mainly in urine (Lee et al., 1995).

### Toxicity and Limitations of Caffeic Acid

CA has exhibited selective toxicity in HCC (Brautigan et al., 2018). In a study, a group of 15 male hamsters was supplemented with 1% (10 g/kg diet) CA (98% pure) for 5 months. Later, the urinary bladders and stomach were examined by histopathology and radiography (Shikov et al., 2014). Moderate epithelial hyperplasia was detected in 14 animals, 1 was severe, and 7 were untreated. An increase in the number of labeled cells was detected in the forestomach and pyloric region when H-thymidine incorporation was assessed in the infected hamsters. It is not statistically significant.

Another study revealed carcinogenic activity in male and female F344 rats and C57BL/6N × C3H/HeN F1 mice in the squamous cell epithelium in the forestomach (Hagiwara et al., 1991). However, successful combinations of potent therapeutic drugs/agents with products may attain the desired conclusion but with lesser toxicity (Bukowska et al., 2015; Das et al., 2018). CA has been believed to be a toxic compound of anaerobic wastewater treatment (Hernandez and Edyvean, 2018). However, CA can act differently in terms of digestibility or toxicity in some anaerobic situations—for example, CA undergoes autoxidation in aqueous systems with oxygen draws, resulting in aggregates with adsorptive properties (Rochelle, 2001). CA is carcinogenic towards animals, but no sufficient evidence exists for humans. To date, the accurate mechanism of the toxic effect of CA remains unknown.

## Conclusion and Future Directions

Phytochemicals are presently huge achievements in cancer prevention and treatment as they possess anti-oxidant, anti-proliferative, anti-angiogenic, pro-apoptotic, and anti-tumor properties. CA is a potential chemotherapeutic agent/drug which demonstrates anti-oxidant, anti-inflammatory, anti-proliferative, anti-microbial, and anti-tumor functions and has an anti-oxidant role in normal cells and a pro-oxidant role in tumor cells. It leads to pro-oxidant-induced oxidative DNA injury, and its downstream pathway stimulates apoptotic tumor cell death. The anti-tumor action of CA appears to be correlated with its promising anti-oxidant and pro-oxidant action attributed to its chemical structure and free phenolic hydroxyls. CA denotes a potent anti-tumor effect in several tumor cells and thus might be used as an anti-tumor agent.

CA plays a central role in preventing cancer development through reduced cell viability and apoptosis induction. CA treatment leads to cell cycle modulation, prevention of cancer progression, and alteration of caspase expression. However, CA stimulates apoptosis by blocking Bcl-2 action, which leads to liberating cyt-*c* and the consequent activation of caspase-3, showing that CA stimulates apoptosis through the intrinsic apoptotic pathway. CA reveals action against numerous cancers, inhibiting the exaggerated creation of ROS and supporting cancer cell destruction *via* DNA oxidation and angiogenesis by acting to decrease VEGF-mediated vascularization and repression of MMP-2 and MMP-9. The repressive result of CA on MMP-2 and MMP-9 is connected with the obstruction of NF-κB activation as verified in cancer cells induced *via* PMA, which reduces cancer invasiveness and growth.

The anti-carcinogenic action of CA has been established, and the mechanism of action has been extensively studied. This opens an important scope for prospective investigations of CA in combination with chemical moieties/drugs in authorized animal models. Such studies might lead to the improvement of CA as a potential clinical aspirant in diverse cancer circumstances as combination therapy. CA, both in its free appearance and when conjugated with other moieties, generates its considerable pharmacological effect.

Combination therapy is an attractive alternative to drug development in pharmaceutical manufacturing to resolve drug resistance, decrease unfavorable drug reactions, and enhance drug efficiency. Multiple disease situations primarily need a combination therapy because of their difficult pathophysiology and progression. However, drug development research needs more data on suitable efficacy for a superior translational result in clinical trials.

CA possesses a strong anti-cancer effect and can be an effective chemotherapeutic agent. Further detailed clinical trials on CA will be significant in developing novel anti-cancer drugs. The experimental and clinical findings reveal the various anti-tumor properties of CA against several cancers, which might sensitize cancer cells and decrease cancer growth and survival. CA has a broad range of biological activities. However, CA alone or in combination with other chemotherapeutic agents/drugs might be suggested to treat and manage cancer.

## Abbreviations

AAA, aromatic amino acids; CA, caffeic acid; CAPE, caffeic acid phenethyl ester; CGA, chlorogenic acid; CGA, chlorogenic acid; CLL, chronic lymphocytic leukemia; COX-2, cyclooxygenase 2; ECM, extracellular matrix; EGFR, epidermal growth factor receptor; ERK, extracellular signal-regulated kinase; H_2_O_2_, hydrogen peroxide; HCs, hydroxycinnamic acids; JNK, c-Jun N-terminal kinase; MCT, monocarboxylic acid transporters; MMP, matrix metalloproteinases; NAD, nicotinamide adenine dinucleotide; NF-κB, nuclear factor kappa B; PKC, kinase C protein; PLP, pyridoxal 5-phosphate; ROS, reactive oxygen species; STAT3, transcription factor and signal translation 3; TAL, tyrosine ammonium lyase; TNF, tumor necrosis factor; VEGF, vascular endothelial growth factor
